# Mind the gap: closure of right to left shunts for rare indications

**DOI:** 10.1007/s12471-018-1164-7

**Published:** 2018-10-04

**Authors:** T. J. F. ten Cate, A. P. J. van Dijk

**Affiliations:** Department of Cardiology, Radboudumc, Nijmegen, The Netherlands

In the current issue of the *Netherlands Heart Journal*, Koopsen et al. [1] describe the mid-term follow-up of a series of divers that suffered from decompression illness (DCI) and assessed how closure of the right-to-left heart shunt influenced the recurrence of DCI.

A patent foramen ovale (PFO) occurs in about 30% of the general population. Under normal conditions the left heart pressures exceed the right heart pressures and the PFO is ‘closed’. However, during increased venous return to the right heart the PFO can become patent. This intermittent right-to-left shunt is thought to be associated with various conditions [2–5]. Although PFO closure has been extensively studied for all of the above-mentioned conditions, closure for all indications remains a controversial topic.

For divers, the presence of a right-to-left shunt carries an increased risk for DCI [1, 5]. Although the incidence of DCI is low, there is a 5-fold increased risk in the presence of a right-to-left shunt [5]. As DCI is a potentially life-threatening condition, this right-to-left shunt may even be a contra-indication to diving. Therefore, a better understanding of treatment options and risks that these options carry is of utmost importance.

In a previous study in which patients with a PFO and DCI were followed [5], the recurrence of DCI after PFO closure was extremely low compared to those patients who chose not to have the PFO closed. However, the number of closed PFOs was very low.

In this series of 62 divers, a right-to-left shunt was found in 56%. When there was no other contra-indication to diving, patients with a PFO or an atrial septal defect (ASD) were offered percutaneous closure, which was subsequently performed in 21 (60%) patients. The treated patients all had a follow-up echocardiogram that revealed complete closure of the PFO/ASD.

In the group with a closed PFO/ASD unrestricted diving was allowed 6 months after closure. The majority continued diving, most of them without restrictions. These patients did not suffer from a recurrent DCI during follow-up (mean 6.8 years). This finding differs from the group in whom the PFO was not closed. These patients had various reasons not to close the PFO, either personal or clinical, that made PFO closure futile. Two patients in the small group that continued diving suffered from minor decompression sickness.

Because this patient population is small and the implications of DCI are large, it is important to better understand the options in currently available treatment. As not all of the patients in the current study underwent closure, we now better understand the consequences of the options. The authors report that the recurrence of DCI during follow-up was very low, even when unrestricted diving was restarted. In this study one type of closure device was predominantly used. All patients underwent echocardiographic confirmation of the absence of residual shunt.

Until now the presence of a right-to-left shunt more or less meant the end of diving, whether recreational or professional. The current study enhances our understanding of the risks and options of diving with a right-to-left shunt and allows for better counselling of the patient. The data indicate that refraining from diving remains the safest option. However, the current study shows that, after PFO/ASD closure, diving without restriction is feasible and relatively safe. Nevertheless, when patients choose not to have the PFO/ASD closed, the option of restricted diving remains, even though the risk of potentially life-threatening DCI remains high.

The findings of the current study by Koopsen et al. [1] indicate that it may be time to change our opinions about diving in the presence of a right-to-left shunt. Their results clearly demonstrate that it may be time for a paradigm shift. In the present era of more demanding patients, the flow chart provided in the current edition of the *Netherlands Heart Journal* is a clear and useful tool to use when presented with a patient with PFO/ASD who has suffered from DCI. Perhaps even preventive closure in people who have a clear and present desire to dive comes into play. However for this patient population, and also for patients with other indications of right-to-left shunt closure, it is important to understand whether all devices are equal or that some are more equal than others.
